# A multi-predictor nonparametric local linear generalized Poisson regression model for overdispersed count data^,^^☆^^☆☆^

**DOI:** 10.1016/j.mex.2026.104228

**Published:** 2026-10-23

**Authors:** Darnah, M. Fathurahman, Iriyani Kamaruddin, Suyitno, Toha Saifudin, Mardiana Idayu Ahmad, Erni Tri Astuti, Ratna Kusuma, Rahmawati Munir, Made Dirgantara, Fidia Deny Tisna Amijaya, Imam Rofi’i, Andi Syaifullah Al Maslulil Agri, Feni Arista Bahni

**Affiliations:** a Statistics Study Program, Department of Mathematics, Faculty of Mathematics and Natural Sciences, Mulawarman University, Samarinda, Indonesia; b Nutrition Study Program, Faculty of Public Health, Mulawarman University, Samarinda, Indonesia; c Statistics Study Program, Department of Mathematics, Faculty of Sciences and Technology, Airlangga University, Surabaya, Indonesia; d School of Industrial Technology, Universiti Sains Malaysia, 11800 Penang, Malaysia; e Polytechnic of Statistics STIS, Jakarta, Indonesia; f Biology Study Program, Department of Biology, Faculty of Mathematics and Natural Sciences, Mulawarman University, Samarinda, Indonesia; g Physics Study Program, Department of Physics, Faculty of Mathematics and Natural Sciences, Mulawarman University, Samarinda, Indonesia; h Physics Study Program, Faculty of Mathematics and Natural Sciences, Palangka Raya University, Palangka Raya, Indonesia; i Mathematics Study Program, Department of Mathematics, Faculty of Mathematics and Natural Sciences, Mulawarman University, Samarinda, Indonesia; j Universitas Muhammadiyah Kalimantan Timur, Samarinda, Indonesia; k Environmental Science Study Program, Department of Biology, Faculty of Mathematics and Natural Sciences, Mulawarman University, Samarinda, Indonesia

**Keywords:** Nonparametric regression, Local linear estimator, Generalized Poisson distribution, Overdispersed, Count data

## Abstract

Generalized Poisson regression is widely used for modeling overdispersed count data; however, existing nonparametric local linear generalized Poisson regression methods are generally restricted to a single predictor, limiting their applicability to datasets involving multiple predictors. This study extends the nonparametric local linear generalized Poisson regression framework to accommodate multiple predictors, namely multi-predictor local linear generalized Poisson regression (MP-LLGPR). The proposed method provides a flexible approach for modeling overdispersed count response variable with locally varying relationships involving multiple predictors. The method is empirically validated in a healthcare application using stunting count data from 44 regencies and cities in Kalimantan, Indonesia. The empirical validation demonstrates the applicability of the proposed method for modeling overdispersed count data with multiple predictors and provides a comparison with the corresponding global generalized Poisson regression model.

## Highlights

• Develops a multi-predictor extension of nonparametric local linear generalized Poisson regression model.
• Implements leave-one-out cross-validation (CV) based bandwidth selection for data-driven local estimation.
• The method is empirically validated in healthcare application using stunting count data in Kalimantan, Indonesia.

## Graphical abstract

**Figure:**
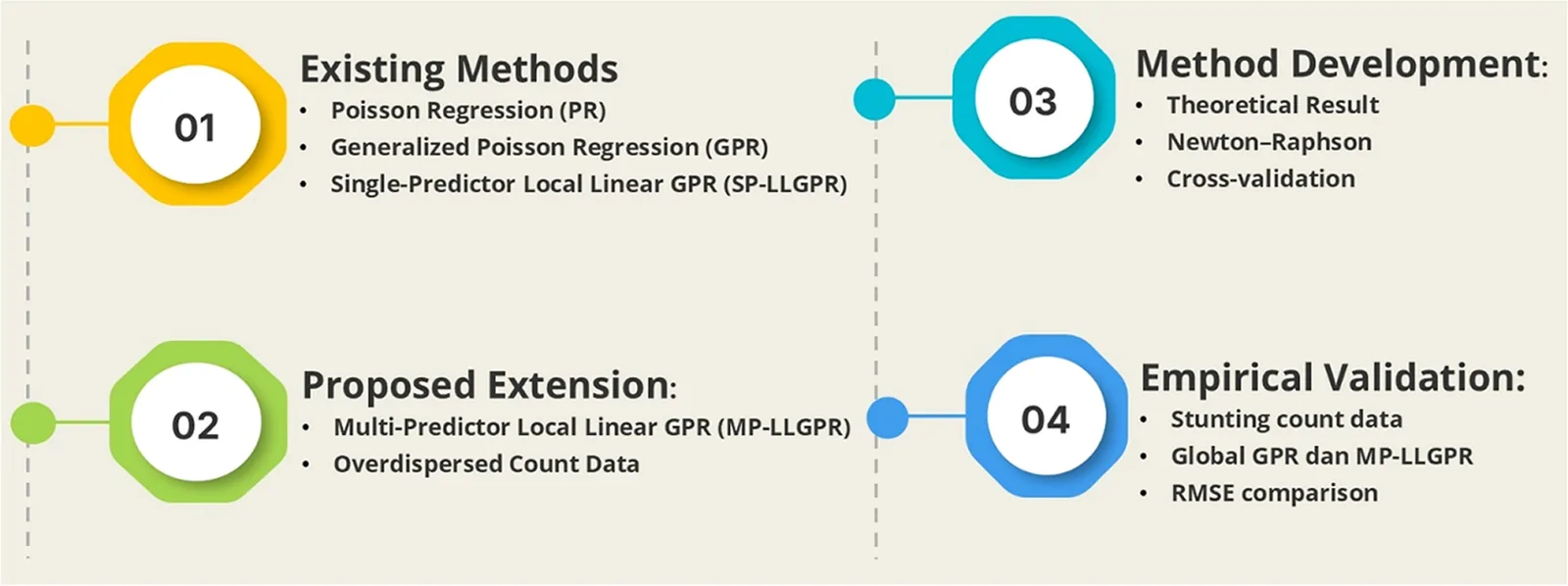

Specifications table

**Table:** 

**Subject area**	Mathematics and Statistics
**More specific subject area**	Statistical Modeling; Nonparametric Regression; Generalized Poisson Regression; Local Linear Regression; Overdispersed Count Data.
**Name of your method**	Multi-predictor Nonparametric Local Linear Generalized Poisson Regression (MP-LLGPR)
**Name and reference of original method**	Darnah, Memi Nor Hayati, Sri Wahyuningsih, Iriyani Kamaruddin, Suyitno, Andrea Tri Rian Dani, Rito Goejantoro, Desi Yuniarti, Fidia Deny Tisna Amijaya, Ika Purnamasari, Meiliyani Siringoringo, Surya Prangga, Ratna Kusuma, Rahmawati Munir, "Locally kernel weighted maximum likelihood estimator for local linear multi-predictor poisson regression", MethodsX, 14(103,258), 2025, https://doi.org/10.1016/j.mex.2025.103258. Darnah, M. I. Utoyo, and N. Chamidah, “Modeling of Maternal Mortality and Infant Mortality Cases in East Kalimantan using Poisson Regression Approach Based on Local Linear Estimator,” in IOP Conference Series: Earth and Environmental Science, Institute of Physics Publishing, Apr. 2019. doi:10.1088/1755–1315/243/1/012,023. D. Darnah, M. Imam Utoyo, and N. Chamidah, “Estimation of the Bi-response Poisson Regression Model Based on Local Linear Approach,” International Journal of Academic and Applied Research, vol. 3, no. 5, pp. 14–18, 2019, [Online]. Available: http://www.ijeais.org/ijaar Darnah, I. Utoyo, and N. Chamidah, “Bi-response Poisson regression model for modeling effect of early marriage on maternal and infant mortality,” AIP Conf Proc, vol. 2554, no. 1, p. 030,005, Jan. 2023, doi: 10.1063/5.0104404.
**Resource availability**	None

## Background

Poisson regression is one of the most widely used statistical methods for modeling count data because it relates the expected count to a set of explanatory variables through a log-link function. However, the classical Poisson regression model relies on the equidispersion assumption, in which the mean and variance of the response variable are equal. In practice, this assumption may be violated because of overdispersion or underdispersion, which can affect parameter estimation, standard errors, and statistical inference [[1], [2], [3], [4], [5], [6], [7], [8]] . To address this limitation, Wang and Famoye [9] introduced the generalized Poisson regression (GPR) model, which extends the classical Poisson regression by incorporating a dispersion parameter and provides a flexible framework for modeling equidispersed, overdispersed, and underdispersed count data.

Regression models can be developed using either parametric or nonparametric approaches. Parametric regression assumes a predetermined functional form for the relationship between the response and predictor variables [[10], [11], [12], [13], [14], [15], [16], [17], [18]] . However, this assumption may be restrictive when the underlying relationships are complex or nonlinear. Nonparametric regression provides a flexible alternative by estimating the regression function from the observed data without imposing a specific parametric functional form [[19], [20], [21]].

Various smoothing approaches have been developed for nonparametric regression, including kernel, spline, penalized spline, truncated spline, local polynomial, and local linear estimators [[21], [22], [23], [24], [25], [26], [27], [28], [29], [30], [31], [32], [33], [34], [35], [36], [37], [38], [39], [40], [41], [42], [43]] . Among these approaches, local linear estimation provides a flexible mechanism for approximating an unknown regression function locally and has been applied to Poisson regression models for modeling nonlinear relationships between count responses and predictor variables [1,2,11,12,35,44].

The local linear approach has also been extended to generalized Poisson regression to accommodate nonlinear relationships in overdispersed count data. Astuti [22,45] developed a local likelihood-based generalized Poisson regression approach; however, the formulation is restricted to a single predictor variable, limiting its applicability when the response is simultaneously associated with multiple predictors. In addition, the existing approach determines the smoothing parameter using the root mean square error (RMSE), whereas bandwidth selection can instead be formulated as a cross-validation problem. These limitations motivate an extension of the local linear generalized Poisson regression framework to accommodate multiple predictors and to use a data-driven cross-validation criterion for bandwidth selection.

Accordingly, this study develops a MP-LLGPR method for modeling overdispersed count data. The proposed extension incorporates multiple predictors within a local linear generalized Poisson framework, while bandwidth selection is performed using CV. The method provides a unified framework for locally estimating nonlinear relationships between an overdispersed count response and multiple predictors. Its empirical performance is validated using stunting count data from 44 regencies and cities in Kalimantan, Indonesia. The stunting dataset used to demonstrate the applicability and model performance of the proposed method rather than as a substantive analysis of determinants of stunting.

## Method details

### Poisson regression model

The response variable in a Poisson regression model is assumed to follow a Poisson distribution, which belongs to the exponential family of distributions. This model is classified as a generalized linear model consisting of three components, that is random component, systematic component, and link function. Let Ybe the random variable for count data and it follows a Poisson distribution, the probability density function (PDF) is

(1) $$P\left(Y=y|\theta \right)=f\left(y,\theta \right)=\frac{\theta^{y}\exp\left(-\theta \right)}{y!},\;\;y=0,\;1,\;2,\;\ldots$$

with mean and variance,

(2) E(Y)=Var(Y)=θ

If Eq. (1) rewritten in the form of the PDF of the exponential family of distributions, then [46]

(3) $$f\left(y,\theta \right)=exp[y\log\left(\theta \right)-\theta -\log\left(y_{i}!\right)$$

where Yis a random component, E(Y)=θ, and g(θ)=log(θ) is canonical link function. The link function relates the response variable Y and the k predictors X, yielding the Poisson regression model as follows:

(4) $$\theta_{i}=\exp\left(x_{i}^{T}\beta \right)$$

with $x_{i}^{T}=\left[\begin{array}{ccccc} 1 & x_{1i} & x_{2i} & \ldots & x_{ki} \end{array}\right]$ is the i th observation result data vector of the predictor variables and $\beta ={\left[\begin{array}{ccccc} \beta_{0} & \beta_{1} & \beta_{2} & \ldots & \beta_{k} \end{array}\right]}^{T}$. Poisson regression model given by Eq. (4) is nonlinear regression [47]. A commonly used parameter estimation method for nonlinear regression models, such as Weibull regression model [48,49] and ordinal logistic regression model [50], is maximum likelihood estimation (MLE) by maximizing likelihood function. The likelihood function in Eq. (1) is defined as

(5) $$\ell \left(\beta \right)=\prod_{i=1}^{n}f(y_{i},\theta_{i})=\prod_{i=1}^{n}\frac{\exp[y_{i}x_{i}^{T}\beta -\exp\left(x_{i}^{T}\beta \right)]}{y_{i}!}$$

and the log-likelihood function is

(6) $$L\left(\beta \right)=log\left(l\left(\beta \right)\right)=\sum_{i=1}^{n}(y_{i}x_{i}^{T}\beta -\exp\left(y_{i}x_{i}^{T}\beta \right)-\log\left(y_{i}!\right)$$

The vector β can be obtained by maximizing log-likelihood function in Eq. (6) and setting its first derivative equal to zero, yielding

(7) $$\frac{\partial L(\beta )}{\partial \beta }=0$$

Since Eq. (7) has no closed-form analytical solution, a numerical method is required to obtain the maximum likelihood estimator. The Newton-Raphson iterative algorithm is commonly used for this purpose.

### Generalized Poisson regression model

According to Ismail and Jemain [8], let $(Y_{i},x_{i}),i=1,\;2,\;\cdots ,\;n$ be a count random variable following a generalized Poisson distribution, the corresponding probability density function is defined as

(8) $$f\left(y;\theta ,\varphi \right)={\left(\frac{\theta }{1+\varphi \theta }\right)}^{y}\frac{{\left(1+\varphi y\right)}^{y-1}}{y\;!}\exp\left(-\frac{\theta (1+\varphi y)}{1+\varphi \theta }\right);y=0,\;1,\;2,\cdots$$

The mean value in generalized Poisson distribution is

(9) E(Y)=θ

and the variance is

(10) $$\text{Var}\left(Y\right)=\theta {\left(1+\varphi \theta \right)}^{2}$$

The parameter φ in Eq. (8) represents the dispersion parameter. When φ=0, the model reduces to the Poisson distribution, when φ<0, it indicates underdispersion, and when φ>0, it indicates overdispersion relative to Poisson distribution [23,47]. The mean of the response variable E(Y|x)=θ(x)in the global generalized Poisson regression is assumed to depend on some predictors through a link function

(11) $$\theta \left(x\right)=\exp\left(x^{T}\beta \right)$$

whereβ is the parameter vector of the regression model. The parameters are estimated using MLE. Based on Eq. (8), the likelihood function can be expressed

(12) $$\ell \left(\theta ,\varphi ,y_{i}\right)=\prod_{i=1}^{n}{\left(\frac{\theta }{1+\varphi \theta }\right)}^{y_{i}}\frac{{\left(1+\varphi y_{i}\right)}^{y_{i}-1}}{y_{i}\;!}\exp\left(-\frac{\theta (1+\varphi y_{i})}{1+\varphi \theta }\right)$$

and log-likelihood function is

(13) $$L\left(x^{T}\beta ,\varphi ,y_{i}\right)=\sum_{i=1}^{n}\left\{y_{i}\ln\left(\frac{x_{i}^{T}\beta }{1+\varphi x_{i}^{T}\beta }\right)+\left(y_{i}-1\right)\ln\left(1+\varphi y_{i}\right)-\ln\left(y_{i}\;!\right)-\frac{x_{i}^{T}\beta \left(1+\varphi y_{i}\right)}{1+\varphi x_{i}^{T}\beta }\right\}$$

The maximum likelihood estimator for βand φ are obtained by solving the maximum likelihood equations.

(14) $$\frac{\partial L}{\partial \beta }=\sum_{i=1}^{n}\frac{y_{i}-exp\left(x_{i}^{T}\beta \right)}{{\left(1+\varphi exp\left(x_{i}^{T}\beta \right)\right)}^{2}}=0$$

(15) $$\frac{\partial L}{\partial \varphi }=\sum_{i=1}^{n}\left\{\frac{-y_{i}exp\left(x_{i}^{T}\beta \right)}{1+\varphi exp(x_{i}^{T}\beta )}+\frac{y_{i}\left(y_{i}-1\right)}{1+\varphi y_{i}}-\frac{exp\left(x_{i}^{T}\beta \right)\left(y_{i}-exp\left(x_{i}^{T}\beta \right)\right)}{{\left(1+\varphi exp\left(x_{i}^{T}\beta \right)\right)}^{2}}\right\}=0$$

Since Eq. (14) and (15) are nonlinear in parameters, the solution can be obtained by using some iterative methods such as Newton Raphson.

Nonparametric local linear generalized Poisson regression model for a predictor variable

In the nonparametric local linear generalized Poisson regression model, rather than assuming a specific regression function, the relationship between the mean response and a predictor variable is represented by a smooth nonparametric regression function as follows:

(16) $$E\left(Y_{i}|x\right)=\theta_{i}\left(x\right)=\exp\left[m\left(x_{i}\right)\right]$$

Suppose m is a continuous function that has a (p+1)-th derivative at the point $x_{0}$. Then, for any point $x_{i}$in the neighborhood of $x_{0}$, the function m can be expanded into the following Taylor series of degree p.

(17) $$m\left(x_{i}\right)\approx m\left(x_{0}\right)+m^{\prime }\left(x_{0}\right)\left(x_{i}-x_{0}\right)+\frac{m^{\prime \prime }\left(x_{0}\right){\left(x_{i}-x_{0}\right)}^{2}}{2!}+\cdots +\frac{m^{\left(p\right)}\left(x_{0}\right){\left(x_{i}-x_{0}\right)}^{p}}{p!}=x_{i}^{*T}\beta$$

where $x_{i}^{*}={\left[\begin{array}{ccccc} 1 & \left(x_{i}-x_{0}\right) & {\left(x_{i}-x_{0}\right)}^{2} & \ldots & {\left(x_{i}-x_{0}\right)}^{p} \end{array}\right]}^{T}$, $\beta ={\left[\begin{array}{ccccc} \beta_{0} & \beta_{1} & \beta_{2} & \ldots & \beta_{p} \end{array}\right]}^{T}$, with $\beta_{\nu }=s^{\nu }\left(x_{0}\right)/\nu !$ and ν=0,1,2,…,p.

The regression curve at point $x_{0}$ in nonparametric regression is estimated using the locally kernel weighted maximum likelihood estimator method, in which the model parameters are first estimated. In Eq. (17), if p = 0, the technique is called local constant and if p = 1, it is known as local linear model [23].

Based on Eq. (8) and (17), we obtain the local kernel weighted log-likelihood function

(18) $$L_{p}\left(x_{i}^{*T}\beta ,\varphi ,y_{i}\right)=\sum_{i=1}^{n}K_{h}\left(x_{i}-x_{0}\right)\left\{y_{i}\ln\left(\frac{x_{i}^{*T}\beta }{\left(1+\varphi x_{i}^{*T}\beta \right)}\right)+\left(y_{i}-1\right)\ln\left(1+\varphi y_{i}\right)-\ln\left(y_{i}!\right)-\frac{x_{i}^{*T}\beta \left(1+\varphi y_{i}\right)}{1+\varphi x_{i}^{*T}\beta }\right\}$$

where K is kernel function and h > 0 represent the bandwidth. The bandwidth h controls smoothness of the fit curve. If h is too small, the fit becomes too noisy, and the variance increases. Conversely, if h is too large, the fit becomes over-smoothed and important feature may be distorted or lost completely. That is, the fit will have large bias. The bandwidth must be chosen to compromise this bias-variance trade-off. The CV method is a commonly used technique to determine the optimal bandwidth by minimizing the CV value.

Based on Eq. (18), the estimators βand φ are obtained by solving the (p + 2) equations.

(19) $$\frac{\partial L_{p}}{\partial \beta }=\sum_{i=1}^{n}K_{h}\left(x_{i}-x_{0}\right)\frac{y_{i}-\exp\left(x_{i}^{*T}\beta \right)}{{\left(1+\varphi \exp\left(x_{i}^{*T}\beta \right)\right)}^{2}}x_{i}=0$$

(20) $$\frac{\partial L_{p}}{\partial \varphi }=\sum_{i=1}^{n}K_{h}\left(x_{i}-x_{0}\right)\left\{\frac{-y_{i}\exp\left(x_{i}^{*T}\beta \right)}{1+\varphi \exp(x_{i}^{*T}\beta )}+\frac{y_{i}\left(y_{i}-1\right)}{1+\varphi y_{i}}-\frac{\exp\left(x_{i}^{*T}\beta \right)\left(y_{i}-\exp\left(x_{i}^{*T}\beta \right)\right)}{{\left(1+\varphi \exp\left(x_{i}^{*T}\beta \right)\right)}^{2}}\right\}=0$$

The solutions to Eqs. (19) and (20) can be obtained using the Newton–Raphson method.

Proposed multi-predictor nonparametric local linear generalized Poisson regression (MP-LLGPR) model

Let $\left(y_{i},x_{i}\right)$,i=1,2,⋯,n be the observed data pairs, where $y_{i}$ is the count response that follows the generalized Poisson distribution and $x_{i}=\left(\begin{array}{ccccc} x_{i1} & x_{i2} & x_{i3} & \ldots & x_{ik} \end{array}\right)$ is a vector of k predictor variables. The dependence of the mean response on the k predictors is described by a smooth nonparametric regression function

(21) $$\theta \left(x_{i}\right)=\exp\left[m\left(x_{i}\right)\right]$$

where $m(x_{i})$ is an unknown function, from the exponential family function, and continuous function that has a (p+1)-th derivative at the point $x_{0}$that we aim to estimate using locally kernel weighted maximum likelihood estimator method.

The assigned weights are determined by the relative distances between the observed predictor values and the target point $x_{0}$, such that observations closer to $x_{0}$ receive larger weights than those farther away. The kernel-weighted likelihood function at the point $x_{0}$, which depends on the polynomial degree p and the bandwidth [2], can be expressed

(22) $$\ell \left(\beta ,\varphi ;x_{0}\right)=\prod_{i=1}^{n}f{\left(y_{i};\theta \left(x_{i}\right),\varphi \right)}^{\prod_{j=1}^{k}K_{h_{j}}\left(x_{ij}-x_{0j}\right)}$$

where $h_{j}$denotes the bandwidth associated with the j-th predictor. By approximating $m(x_{i})$using a first-order Taylor expansion, we obtain

(23) $$m\left(x_{i}\right)=\beta_{0}+\sum_{j=1}^{k}\beta_{j}\left(x_{ij}-x_{0j}\right)$$

Eq. (23) can be expressed in vector form as follows

(24) $$m\left(x_{i}\right)=x_{i}^{*T}\beta$$

where βis the vector of local regression coefficients. The complete parameter vector of the MP-LLGPR model is defined as

(25) $$\psi =\left[\begin{array}{c} \beta \\ \varphi \end{array}\right]={\left(\beta_{0},\beta_{1},\beta_{2},\cdots ,\beta_{k},\varphi \right)}^{T}$$

The local mean parameter is given by $\theta \left(x_{i}\right)=\exp\left(x_{i}^{*T}\beta \right)$. Based on Eq. (21), Eq. (8) can be expressed

(26) $$f\left(y_{i};\theta \left(x_{i}\right),\varphi \right)={\left(\frac{\theta (x_{i})}{1+\varphi \theta (x_{i})}\right)}^{y_{i}}\frac{{\left(1+\varphi y_{i}\right)}^{y_{i}-1}}{y_{i}!}\exp\left(-\frac{\theta \left(x_{i}\right)\left(1+\varphi y_{i}\right)}{1+\varphi \theta (x_{i})}\right)$$

**Definition 1**. Let we have the Kernel-weighted likelihood function in Eq. (22) and the probability density function of $y_{i}$ in Eq. (26) then the local likelihood function can be written as

(27) $$\ell \left(y_{i};\theta \left(x_{i}\right),\varphi \right)={\prod_{i=1}^{n}{\left(\frac{\theta (x_{i})}{1+\varphi \theta (x_{i})}\right)}^{y_{i}}\frac{{\left(1+\varphi y_{i}\right)}^{y_{i}-1}}{y_{i}!}\exp\left(-\frac{\theta \left(x_{i}\right)\left(1+\varphi y_{i}\right)}{1+\varphi \theta (x_{i})}\right)}^{\prod_{j=1}^{k}K_{h_{j}}\left(x_{ij}-x_{0j}\right)}$$

and the local log-likelihood function is

(28) $$\begin{array}{c} L\left(y_{i};\theta \left(x_{i}\right),\varphi \right)=\ln\left(\ell \left(y_{i};\theta \left(x_{i}\right),\varphi \right)\right)=\ln\left(\prod_{i=1}^{n}{\left\{{\left(\frac{\theta (x_{i})}{1+\varphi \theta (x_{i})}\right)}^{y_{i}}\frac{{\left(1+\varphi y\right)}^{y_{i}-1}}{y_{i}\;!}\exp\left(-\frac{\theta \left(x_{i}\right)\left(1+\varphi y_{i}\right)}{1+\varphi \theta (x_{i})}\right)\right\}}^{\prod_{j=1}^{k}K_{h_{j}}\left(x_{ij}-x_{0j}\right)}\right)\\ =\sum_{i=1}^{n}\left(\prod_{j=1}^{k}K_{h_{j}}\left(x_{ij}-x_{0j}\right)\right)\left\{y_{i}\ln\left(\frac{\theta (x_{i})}{1+\varphi \theta (x_{i})}\right)+\left(y_{i}-1\right)\ln\left(1+\varphi y_{i}\right)-\ln\left(y_{i}!\right)\;-\frac{\theta \left(x_{i}\right)\left(1+\varphi y_{i}\right)}{1+\varphi \theta (x_{i})}\right\} \end{array}$$

**Theorem**. Suppose the multi-predictor nonparametric local linear generalized Poisson regression (MP-LLGPR) model specified in Eq. (21), the regression function$m(x_{i})$defined in Eq. (24), and the log-likelihood function presented in Eq. (28) can be expressed $L(y_{i};\theta \left(x_{i}\right),\varphi )=L$, the local weighted maximum likelihood estimators of βand φat the point $x_{0}$ are obtained by solving the following system of equation

(29) $$\frac{\partial L}{\partial \beta }=\sum_{i=1}^{n}\left(\prod_{j=1}^{k}K_{h_{j}}\left(x_{ij}-x_{0j}\right)\right)\left\{\frac{y_{i}-\theta \left(x_{i}\right)}{{\left(1+\varphi \theta \left(x_{i}\right)\right)}^{2}}x_{i}^{*}\right\}=0$$

(30) $$\frac{\partial L}{\partial \varphi }=\sum_{i=1}^{n}\left(\prod_{j=1}^{k}K_{h_{j}}\left(x_{ij}-x_{0j}\right)\right)\left\{-\frac{\left(y_{i}\right)\theta \left(x_{i}\right)}{1+\varphi \theta (x_{i})}+\frac{y_{i}\left(y_{i}-1\right)}{1+\varphi y_{i}}-\frac{\theta \left(x_{i}\right)\left(y_{i}-\theta \left(x_{i}\right)\right)}{{\left(1+\varphi \theta \left(x_{i}\right)\right)}^{2}}\right\}=0$$

Proof. The maximum likelihood estimator for the MP-LLGPR model can be obtained by maximizing the log-likelihood function presented in Eq. (29). The first derivative of Eq. (29) with respect to β using the chain rule can be expressed as

(31) $$\frac{\partial L}{\partial \beta }=\frac{\partial L}{\partial \theta (x_{i})}\frac{\partial \theta (x_{i})}{\partial \beta }$$

where

(32) $$\begin{array}{c} \frac{\partial L}{\partial \theta (x_{i})}=\sum_{i=1}^{n}\left(\prod_{j=1}^{k}K_{h_{j}}\left(x_{ij}-x_{0j}\right)\right)\left\{y_{i}\left(\frac{1+\theta (x_{i})}{\theta (x_{i})}\right)\left(\frac{1}{{\left(1+\varphi \theta \left(x_{i}\right)\right)}^{2}}\right)-\left(1+\varphi y_{i}\right)\left(\frac{1}{{\left(1+\varphi \theta \left(x_{i}\right)\right)}^{2}}\right)\right\}\\ =\sum_{i=1}^{n}\left(\prod_{j=1}^{k}K_{h_{j}}\left(x_{ij}-x_{0j}\right)\right)\left\{\frac{y_{i}-\theta \left(x_{i}\right)}{\theta \left(x_{i}\right){\left(1+\varphi \theta \left(x_{i}\right)\right)}^{2}}\right\} \end{array}$$

and

(33) $$\frac{\partial \theta (x_{i})}{\partial \beta }=\frac{\partial }{\partial \beta }(\exp\left(x_{{}_{i}}^{T}\beta \right)=x_{i}^{*}\theta \left(x_{i}\right)$$

Eq. (32) and (33) substituted into Eq. (31), we obtained

(34) $$\frac{\partial L}{\partial \beta }=\sum_{i=1}^{n}\left(\prod_{j=1}^{k}K_{h_{j}}\left(x_{ij}-x_{0j}\right)\right)\left\{\frac{y_{i}-\theta \left(x_{i}\right)}{{\left(1+\varphi \theta \left(x_{i}\right)\right)}^{2}}x_{i}^{*}\right\}=0$$

Furthermore, the first derivative of Eq. (28) with respect to φ can be expressed as

(35) $$\begin{array}{c} \frac{\partial L}{\partial \varphi }=\sum_{i=1}^{n}\left(\prod_{j=1}^{k}K_{h_{j}}\left(x_{ij}-x_{0j}\right)\right)\left\{-y_{i}\frac{\partial }{\partial \varphi }\ln\left(1+\varphi \theta (x_{i})\right)+\left(y_{i}-1\right)\frac{\partial }{\partial \varphi }\ln\left(1+\varphi y_{i}\right)-\frac{\partial }{\partial \varphi }\frac{1+\varphi y_{i}}{1+\varphi \theta (x_{i})}\right\}\\ =\sum_{i=1}^{n}\left(\prod_{j=1}^{k}K_{h_{j}}\left(x_{ij}-x_{0j}\right)\right)\left\{-\frac{y_{i}\theta \left(x_{i}\right)}{1+\varphi \theta (x_{i})}+\frac{y_{i}\left(y_{i}-1\right)}{1+\varphi y_{i}}-\frac{\theta \left(x_{i}\right)\left(y_{i}-\theta \left(x_{i}\right)\right)}{{\left(1+\varphi \theta \left(x_{i}\right)\right)}^{2}}\right\}=0 \end{array}$$

Eq. (34) and (35) are implicit and therefore require an iterative procedure to obtain their solutions. In this study, the Newton–Raphson iterative method [41] is employed to estimate the parameters.

**Definition 2.** Let the first derivatives of the local log-likelihood function with respect to the parameter$\beta ={\left(\beta_{0},\beta_{1},\beta_{2},\cdots ,\beta_{k}\right)}^{T}$and φ,as shown in Eq. (34) and (35), $x_{ij};\;\;j=1,2,...,k$ is predictor variable, the gradient vector is

(36) $$g\left(\psi \right)=\frac{\partial L}{\partial \psi }=\left[\begin{array}{c} \frac{\partial L}{\partial \beta }\\ \frac{\partial L}{\partial \varphi } \end{array}\right]$$

and the Hessian matrix is

(37) $$H\left(\psi \right)=\frac{\partial^{2}L}{\partial \psi \partial \psi^{T}}=\left[\begin{array}{cc} H_{\beta \beta } & H_{\beta \varphi }\\ H_{\varphi \beta } & H_{\varphi \varphi } \end{array}\right]$$

The elements of the Hessian matrix in Eq. (37) can be expressed

(38) $$H_{\beta \beta }=\frac{\partial^{2}L}{\partial \beta \partial \beta^{T}}=-\sum_{i=1}^{n}\left(\prod_{j=1}^{k}K_{h_{j}}\left(x_{ij}-x_{0j}\right)\right)\theta \left(x_{i}\right)\left\{\frac{1}{{\left(1+\varphi \theta \left(x_{i}\right)\right)}^{2}}+\frac{2\varphi (y_{i}-\theta \left(x_{i}\right)}{{\left(1+\varphi \theta \left(x_{i}\right)\right)}^{3}}x_{i}^{*}x_{{}_{i}}^{*T}\right\}$$

(39) $$H_{\varphi \varphi }=\frac{\partial^{2}L}{\partial \varphi^{2}}=\sum_{i=1}^{n}\left(\prod_{j=1}^{k}K_{h_{j}}\left(x_{ij}-x_{0j}\right)\right)\left\{\frac{y_{i}{\left(\theta \left(x_{i}\right)\right)}^{2}}{{\left(1+\varphi \theta \left(x_{i}\right)\right)}^{2}}-\frac{y_{{}_{i}}^{2}\left(y_{i}-1\right)}{{\left(1+\varphi y_{i}\right)}^{2}}+\frac{2{\left(\theta \left(x_{i}\right)\right)}^{2}\left(y_{i}-\theta \left(x_{i}\right)\right)}{{\left(1+\varphi \theta \left(x_{i}\right)\right)}^{3}}\right\}$$

(40) $$H_{\beta \varphi }=\frac{\partial^{2}L}{\partial \beta \partial \varphi }=-2\sum_{i=1}^{n}\left(\prod_{j=1}^{k}K_{h_{j}}\left(x_{ij}-x_{0j}\right)\right)\left\{\frac{\theta \left(x_{i}\right)(y_{i}-\theta \left(x_{i}\right)}{{\left(1+\varphi \theta \left(x_{i}\right)\right)}^{3}}x_{i}^{*}\right\}$$

(41) $$H_{\varphi \beta }=H_{\beta \varphi }^{T}$$

The Fisher information matrix is constructed as follows

(42) $$I\left(\psi \right)=-E\left[\frac{\partial^{2}L}{\partial \psi \partial \psi^{T}}\right]$$

Using the gradient vector and Hessian matrix obtained from Eq. (36) and (37), the Newton–Raphson iterative procedure is employed to estimate the model parameters.

(43) $$\psi^{\left(m+1\right)}=\psi^{\left(m\right)}-H^{-1}(\psi^{\left(m\right)})g(\psi^{\left(m\right)});m=0,1,2,..$$

The iterative procedure begins with the selection of initial parameter estimates

(44) $${\hat{\psi }}^{\left(0\right)}\left(x_{0}\right)={\left[{\hat{\beta }}_{0}^{\left(0\right)}\left(x_{0}\right),{\hat{\beta }}_{1}^{\left(0\right)}\left(x_{0}\right),\cdots ,{\hat{\beta }}_{k}^{\left(0\right)}\left(x_{0j}\right),{\hat{\varphi }}^{\left(0\right)}\right]}^{T}$$

The initial parameter vector given in Eq. (44) and continues until convergence is achieved, the stopping criterion is $∥{\hat{\psi }}^{\left(m+1\right)}-{\hat{\psi }}^{\left(m\right)}∥\leq \varepsilon$, where ε is a small positive constant. The initial value of the local regression coefficients are obtained from the global Poisson regression model, while the initial dispersion parameter is set to φ=0. Thus, the initial parameter vector is defined as

(45) $${\hat{\psi }}^{\left(0\right)}={\left(\beta_{Poisson}^{T},0\right)}^{T}$$

**Definition 4.** Suppose the multi-predictor local linear generalized Poisson regression (MP-LLGPR) model is specified in Eq. (21), with the local linear predictor given in Eq. (24). For a given bandwidth vector$h={\left(h_{1},h_{2},\cdots ,h_{k}\right)}^{T}$ the CV criterion is defined as

(46) $$CV\left(h\right)=\frac{1}{n}{\sum_{i=1}^{n}\left[y_{i}-{\hat{\theta }}_{-i}\left(x_{i};h\right)\right]}^{2}$$

where ${\hat{\theta }}_{-i}\left(x_{i};h\right)=\exp\left(x_{i}^{T}{\hat{\beta }}_{-i}\right)$denotes the leave-one-out estimate of the mean parameter obtained by excluding the i th observation. The optimal bandwidth vector is obtained by minimizing the CV criterion,

(47) $$h_{opt}=\arg\;\min\;CV\left(h\right)$$

### Method validation

#### Empirical validation

We evaluated the proposed method using empirical validation data on stunting case counts across 44 regencies/cities in Kalimantan, Indonesia, in 2024. The dataset consists of 1 response variable, namely the number of stunting cases (Y), and 6 initial predictor variables, including the number of integrated community-based health service posts or posyandu (X₁), the number of nutrition health workers (X₂), the percentage of monthly per capita food expenditure (X₃), the percentage of the poor population (X₄), the percentage of infants aged less than six months receiving exclusive breastfeeding (X₅), and the number of low birth weight infants (X₆). The full dataset was compiled from the official publication of the Central Bureau of Statistics (BPS) and the Indonesian Ministry of Health. Fig. 1 presents a flowchart illustrating the overall computational and validation methodology of this study.

**Fig. 1:**
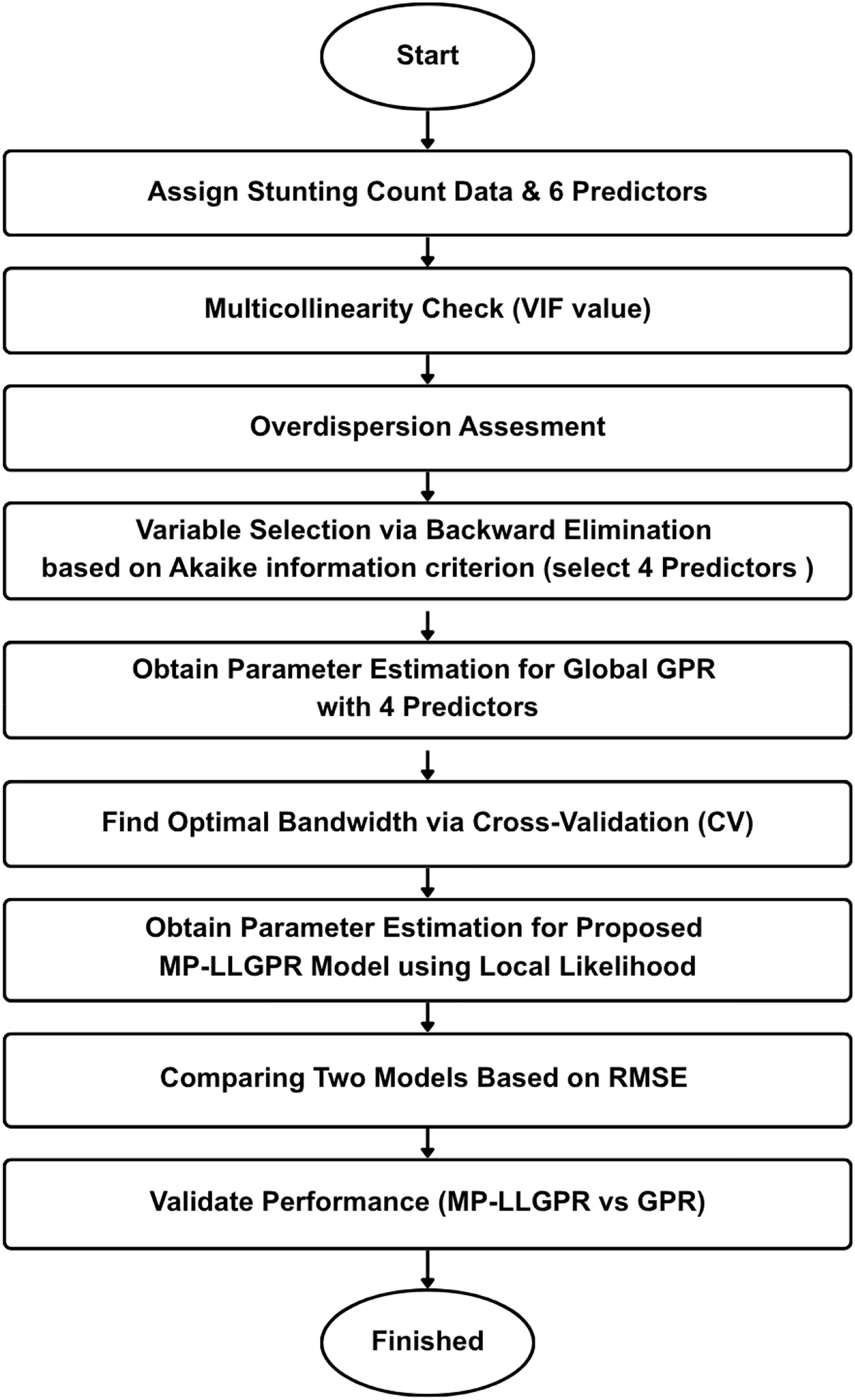
Flowchart of the MP-LLGPR methodology.

Prior to model estimation, descriptive statistical analysis was performed to summarize the initial data characteristics. The resulting descriptive statistics are presented in Table 1. The response variable (Y) displays a mean of 119 and a standard deviation of 43.60 (variance =1900.96), where the sample variance substantially exceeds the mean, indicating initial empirical evidence of overdispersion.

**Table 1:** Descriptive Summary of the Research Variables.

Variable	Average	Minimum	Maximum	Standar Deviation
The stunting cases (Y)	119	31	242	43.60
The number of integrated community-based health service posts or posyandu $\left(X_{1}\right)$	321	36	1688	277.74
The number of nutrition health workers $\left(X_{2}\right)$	58	12	177	33.62
The percentage of monthly per capita food expenditure $\left(X_{3}\right)$	52.22	41.43	61.98	5.14
The percentage of the poor population $\left(X_{4}\right)$	5.66	2.23	10.75	2.00
The percentage of infants aged less than six months receiving exclusive breastfeeding $\left(X_{5}\right)$	64.00	24.80	86.60	14.94
The number of low birth weight infants $\left(X_{6}\right)$	202	0.00	727	168.29

Before fitting the Poisson regression model, potential multicollinearity among the predictor variables was assessed using the variance inflation factor (VIF). The resulting VIF values for all predictor variables are presented in Table 2.

**Table 2:** VIF values of predictor variables.

Variable	VIF value	Decision
The number of integrated community-based health service posts or posyandu $\left(X_{1}\right)$	1.899	No multicollinearity
The number of nutrition health workers $\left(X_{2}\right)$	1.230	No multicollinearity
The percentage of monthly per capita food expenditure $\left(X_{3}\right)$	1.275	No multicollinearity
The percentage of the poor population $\left(X_{4}\right)$	1.097	No multicollinearity
The percentage of infants aged less than six months receiving exclusive breastfeeding $\left(X_{5}\right)$	1.654	No multicollinearity
The number of low birth weight infants $\left(X_{6}\right)$	1.217	No multicollinearity

All predictor variables yielded VIF values below 10 (Table 2), confirming the absence of multicollinearity. Consequently, all six initial predictor variables were included in the full Poisson regression model specification. Parameter estimation for the full Poisson regression model was performed using MLE via the Newton–Raphson algorithm, with the resulting estimates summarized in Table 3.

**Table 3:** Parameter estimates of Poisson regression model using MLE.

Variable	Parameter	Estimate	P-value
Intercept	$\beta_{0}$	2.859	< 2 × 10⁻¹⁶
The number of integrated community-based health service posts or posyandu $\left(X_{1}\right)$	$\beta_{1}$	2.860 × 10⁻⁴	3.42 × 10⁻⁸
The number of nutrition health workers $\left(X_{2}\right)$	$\beta_{2}$	3.452 × 10⁻³	3.46 × 10⁻¹⁰
The percentage of monthly per capita food expenditure $\left(X_{3}\right)$	$\beta_{3}$	2.029 × 10⁻²	3.21 × 10⁻¹¹
The percentage of the poor population $\left(X_{4}\right)$	$\beta_{4}$	4.130 × 10⁻²	1.14 × 10⁻⁷
The percentage of infants aged less than six months receiving exclusive breastfeeding $\left(X_{5}\right)$	$\beta_{5}$	2.296 × 10⁻³	0.0235
The number of low birth weight infants $\left(X_{6}\right)$	$\beta_{6}$	7.577 × 10⁻⁴	3.45 × 10⁻¹⁴

The initial six-predictor Poisson regression model was subsequently assessed for dispersion. The dispersion ratio, calculated as the ratio of the deviance to its residual degrees of freedom, was 9.034, which was substantially greater than 1. This result indicates that the equidispersion assumption of the Poisson model was not satisfied, providing evidence of overdispersion in the stunting case counts. Therefore, the GPR framework was considered appropriate for accommodating the observed dispersion.

To obtain a parsimonious model, backward elimination based on the AIC was applied to the six-predictor GPR model. This variable selection procedure reduced the predictor set from six to four predictors. The selected four-predictor GPR model yielded an AIC value of 438.6039 and was subsequently used as the baseline for developing the proposed MP-LLGPR model. The parameter estimates for the selected four-predictor global GPR model, along with their corresponding significance tests, are summarized in Table 4. All retained predictors were statistically significant at the 5% level, establishing a validated global baseline prior to localized bandwidth optimization and MP-LLGPR model fitting.

**Table 4:** Parameter estimates of the selected global GPR model using MLE.

Variable	Parameter	Estimate	P-value
Intercept	$\beta_{0}$	2.7649	< 0.001
The number of integrated community-based health service posts or posyandu $\left(X_{1}\right)$	$\beta_{1}$	0.0003	0.0442
The number of nutrition health workers $\left(X_{2}\right)$	$\beta_{2}$	0.0037	0.0154
The percentage of monthly per capita food expenditure $\left(X_{3}\right)$	$\beta_{3}$	0.0219	0.0093
The number of low birth weight infants $\left(X_{6}\right)$	$\beta_{6}$	0.0008	0.0044
Dispersion	φ	0.7351	< 0.001
AIC		438.6039	

The optimal bandwidth parameter was determined by minimizing the CV criterion across a specified candidate grid. The resulting bandwidth was applied to the Gaussian kernel function to calculate localized spatial weights for each observation relative to every estimation point. Under this kernel specification, nearby observations receive higher weights, whereas those further away receive exponentially lower weights. The optimal bandwidth and its corresponding CV value are presented in Table 5.

**Table 5:** Optimal bandwidth and CV criterion for the MP-LLGPR model.

Variable	Optimal Bandwidth
The number of integrated community-based health service posts or posyandu $\left(X_{1}\right)$	320
The number of nutrition health workers $\left(X_{2}\right)$	60
The percentage of monthly per capita food expenditure $\left(X_{3}\right)$	9
The number of low birth weight infants $\left(X_{6}\right)$	200
Minimum CV	3546.3172

Based on the optimal bandwidth in Table 5, the parameters of the MP-LLGPR model were estimated . The resulting optimal bandwidth vector was $h_{opt}={\left(320,60,9,200\right)}^{T}$,with a minimum CV value of 3546.3172. The complete set of local maximum likelihood parameter estimates obtained at each observation point, representing the 44 regencies and cities of Kalimantan in 2024, is provided in **Supplementary Table S1**.

To validate the analytical performance and superiority of the proposed MP-LLGPR model over the baseline GPR global model, goodness-of-fit and prediction accuracy were evaluated using RMSE. The final comparative results are summarized in Table 6.

**Table 6:** Performance comparison between global GPR and MP-LLGPR models.

Model	RMSE
GPR global	47.6230
MP-LLGPR	**25.7018**

A comparison based on the RMSE further highlights the improvement achieved by the proposed method (Table 6). The global GPR model produced an RMSE of 47.6230, whereas the MP-LLGPR model yielded a substantially lower RMSE of 25.7018. This represents a 46.03% reduction in RMSE following the incorporation of the localized nonparametric specification. As visually confirmed in Fig. 2, the lower RMSE of the MP-LLGPR model indicates that its fitted values are closer to the observed stunting case counts. These findings demonstrate that allowing parameter estimates to vary locally across the predictor space enables the proposed method to capture heterogeneous relationships between the response variable and multiple predictors across the observed data.

**Fig. 2:**
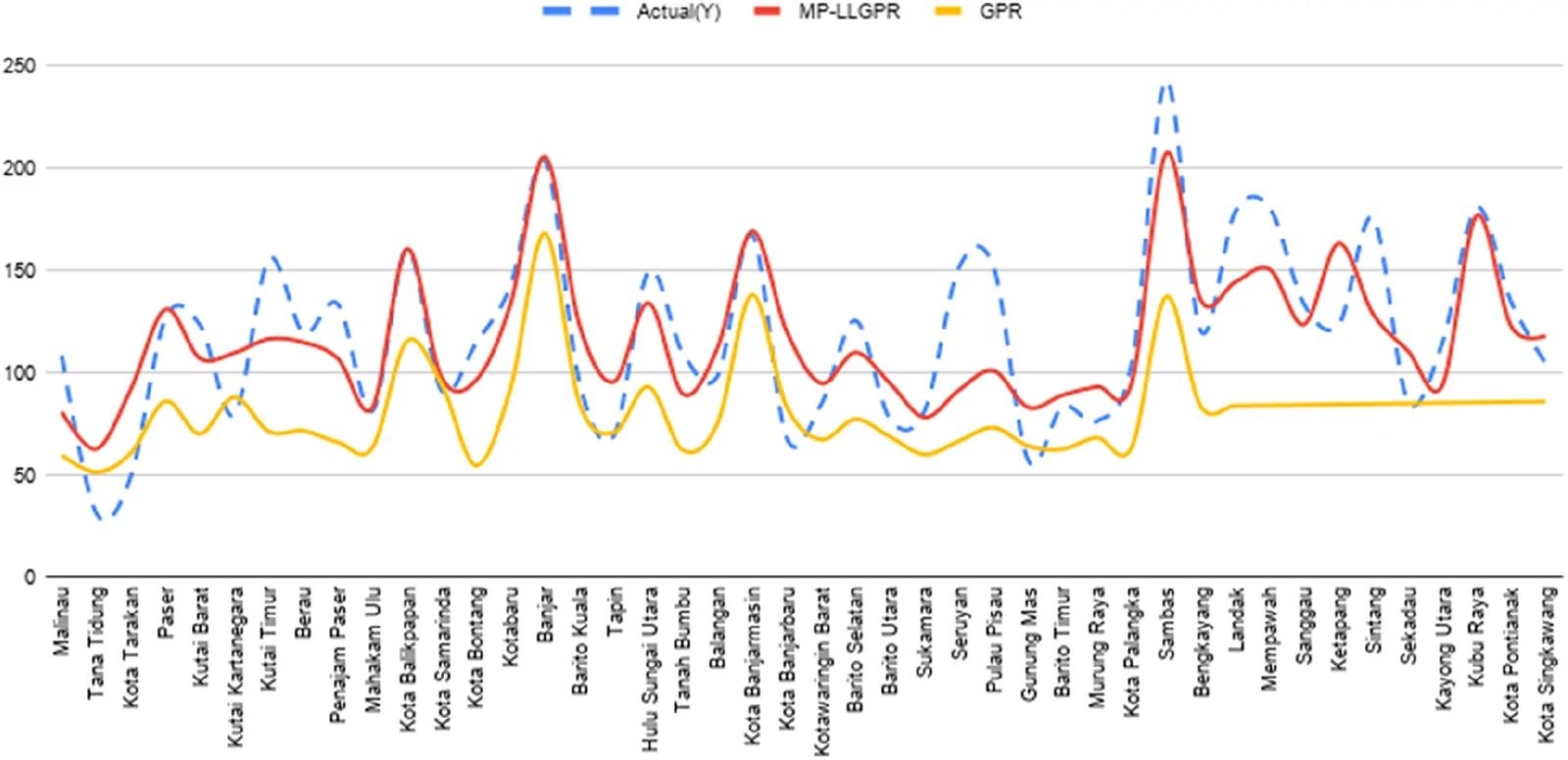
Comparison of observed stunting counts and fitted mean counts from the global GPR and MP-LLGPR models.

### Software

The proposed MP-LLGPR model was implemented in R. The computational procedure included bandwidth selection using a grid search based on leave-one-out CV and local parameter estimation using the Newton–Raphson algorithm. Model performance was evaluated using the RMSE.

## Limitations

The proposed MP-LLGPR method may become computationally demanding as the number of predictors increases because bandwidth optimization is performed in a multidimensional predictor space. In addition, its performance may be affected by the curse of dimensionality when applied to high-dimensional data.

## Ethics statements

This research, entitled “A Multi-predictor Nonparametric Local Linear Generalized Poisson Regression Model for Overdispersed Count Data”, did not involve human participants, animals, or personal data, and thus did not require formal ethical approval. The study focuses on the development and application of statistical methodologies, specifically designed to enhance generalized Poisson regression models using a local linear approach. The research adheres to the highest standards of academic integrity and transparency, with no conflicts of interest, and aims to contribute to the advancement of robust statistical frameworks for health data analysis.

## CRediT author statement

Darnah: Conceptualization, Methodology, Validity test, Writing-Preparation of the first draft, and Supervision. M.Fathurahman: Conceptualization and Methodology. Iriyani Kamaruddin: Methodology and Validity test. Suyitno: Conceptualization, Methodology, and Validity test. Toha Saifudin: Methodology and Validity test. Mardiana: Writing-Review and Editing. Erni Tri Astuti: Methodology and Validity test. Ratna Kusuma: Supervision and Funding Acquisition. Rahmawati Munir: Validity test and Project Administration. Made Dirgantara: Writing-Review and Editing. Fidia Deny Tisna Amijaya: Methodology and Validity test. Imam Rofi’i: Validity test and Project Administration. Andi Syaifullah Al Maslulil Agri: Editing and Project Administration. Feni Arista Bahni: Editing and Project Administration.

**Supplementary material and/or additional information [OPTIONAL]**

None.

## Declaration of competing interest

The authors declare that they have no known competing financial interests or personal relationships that could have appeared to influence the work reported in this paper.

## Data Availability

The authors do not have permission to share data.
