# Supplementary material for: A multi-predictor nonparametric local linear generalized Poisson regression model for overdispersed count data,
Source: MethodsX. 2026 Oct 23;17:104228. doi: 10.1016/j.mex.2026.104228 (PMC13744270; doi:10.1016/j.mex.2026.104228)
Supplement: Supplementary file 1 [file mmc1.docx]

**Supplementary Table S1**. Local parameter estimates of the MP-LLGPR model for all 44 regencies and cities in Kalimantan, Indonesia.

| ***Province*** | ***i*** | ***Regency/City*** | $\hat{\beta}_{0i}$ | $\hat{\beta}_{1i}$ | $\hat{\beta}_{2i}$ | $\hat{\beta}_{3i}$ | $\hat{\beta}_{6i}$ |
| --- | --- | --- | --- | --- | --- | --- | --- |
| *North Kalimantan* | ***1*** | ***Malinau*** | ***4.387893*** | ***0.000755*** | ***0.003126*** | ***0.018859*** | ***0.001451*** |
|  | *2* | *Tana Tidung* | *4.133445* | *0.000973* | *0.003689* | *0.022169* | *0.001617* |
|  | *3* | *Kota Tarakan* | *4.516944* | *0.000854* | *0.002186* | *0.013217* | *0.001144* |
| *East Kalimantan* | *4* | *Paser* | *4.871285* | *0.000543* | *0.002165* | *0.013178* | *0.000690* |
|  | *5* | *Kutai Barat* | *4.669661* | *0.000675* | *0.002345* | *0.013436* | *0.001038* |
|  | *6* | *Kutai Kartanegara* | *4.693970* | *0.000022* | *0.002398* | *0.035663* | *0.000947* |
|  | *7* | *Kutai Timur* | *4.754503* | *0.000585* | *0.001194* | *0.010675* | *0.000789* |
|  | *8* | *Berau* | *4.740101* | *0.000631* | *0.001421* | *0.010793* | *0.000806* |
|  | *9* | *Penajam Paser Utara* | *4.668532* | *0.000701* | *0.002002* | *0.012072* | *0.001040* |
|  | *10* | *Mahakam Ulu* | *4.414171* | *0.000562* | *0.003263* | *0.022180* | *0.001693* |
|  | *11* | *Kota Balikpapan* | *5.075094* | *0.000621* | *0.012379* | *0.026894* | *0.001571* |
|  | *12* | *Kota Samarinda* | *4.579238* | *0.000178* | *0.001618* | *0.031472* | *0.000482* |
|  | ***13*** | ***Kota Bontang*** | ***4.562859*** | ***0.001010*** | ***0.000965*** | ***0.004467*** | ***0.000948*** |
| *South Kalimantan* | *14* | *Kotabaru* | *4.890830* | *0.000543* | *0.003045* | *0.016895* | *0.000746* |
|  | *15* | *Banjar* | *5.322577* | *0.000667* | *0.004292* | *0.040648* | *0.000248* |
|  | *16* | *Barito Kuala* | *4.811111* | *0.000546* | *0.003100* | *0.013764* | *0.000914* |
|  | *17* | *Tapin* | *4.556749* | *0.000552* | *0.003282* | *0.015397* | *0.001419* |
|  | *18* | *Hulu Sungai Utara* | *4.894881* | *0.000544* | *0.002912* | *0.016663* | *0.000691* |
|  | *19* | *Tanah Bumbu* | *4.491036* | *0.000639* | *0.003271* | *0.013961* | *0.001282* |
|  | *20* | *Balangan* | *4.716489* | *0.000594* | *0.002717* | *0.013947* | *0.001056* |
|  | *21* | *Kota Banjarmasin* | *5.127593* | *0.000205* | *0.003812* | *0.033493* | *0.000220* |
|  | ***22*** | ***Kota Banjar Baru*** | ***4.786799*** | ***0.000435*** | ***0.001730*** | ***0.015325*** | ***0.000628*** |
| *Central*  *Kalimantan* | *23* | *Kotawaringin Barat* | *4.548093* | *0.000659* | *0.003107* | *0.015334* | *0.001247* |
|  | *24* | *Barito Selatan* | *4.695934* | *0.000592* | *0.002963* | *0.015834* | *0.001106* |
|  | *25* | *Barito Utara* | *4.547278* | *0.000634* | *0.003063* | *0.016929* | *0.001331* |
|  | *26* | *Sukamara* | *4.354559* | *0.000744* | *0.003225* | *0.021390* | *0.001539* |
|  | *27* | *Seruyan* | *4.512797* | *0.000639* | *0.003028* | *0.017927* | *0.001394* |
|  | *28* | *Pulau Pisau* | *4.611187* | *0.000509* | *0.003067* | *0.017007* | *0.001395* |
|  | *29* | *Gunung Mas* | *4.415392* | *0.000600* | *0.003320* | *0.019007* | *0.001589* |
|  | *30* | *Barito Timur* | *4.485561* | *0.000741* | *0.002848* | *0.016312* | *0.001297* |
|  | ***31*** | ***Murung Raya*** | ***4.531227*** | ***0.000578*** | ***0.003056*** | ***0.018014*** | ***0.001450*** |
|  | *32* | *Kota Palangka Raya* | *4.549889* | *0.000753* | *0.002768* | *0.010061* | *0.000885* |
| *West*  *Kalimantan* | *33* | *Sambas* | *5.330713* | *0.000465* | *0.003373* | *0.033488* | *0.000473* |
|  | *34* | *Bengkayang* | *4.906295* | *0.000560* | *0.002804* | *0.012520* | *0.000637* |
|  | *35* | *Landak* | *4.968176* | *0.000396* | *0.002479* | *0.018978* | *0.000812* |
|  | *36* | *Mempawah* | *5.011160* | *0.000520* | *0.003785* | *0.018968* | *0.000723* |
|  | *37* | *Sanggau* | *4.813505* | *0.000326* | *0.002835* | *0.020654* | *0.001122* |
|  | *38* | *Ketapang* | *5.091898* | *0.000402* | *0.002436* | *0.024183* | *0.000554* |
|  | *39* | *Sintang* | *4.852753* | *0.000400* | *0.003100* | *0.018193* | *0.000989* |
|  | *40* | *Sekadau* | *4.704088* | *0.000437* | *0.003268* | *0.013605* | *0.001300* |
| *West*  *Kalimantan* | *41* | *Kayong Utara* | *4.536967* | *0.000750* | *0.002508* | *0.015955* | *0.001230* |
|  | *42* | *Kubu Raya* | *5.171593* | *0.000314* | *0.002419* | *0.033181* | *0.000541* |
|  | ***43*** | ***Kota Pontianak*** | ***4.805209*** | ***0.000163*** | ***0.002598*** | ***0.019784*** | ***0.000421*** |
|  | *44* | *Kota Singkawang* | *4.768516* | *0.000635* | *0.002351* | *0.014952* | *0.000683* |
